# The effects of ketogenic metabolic therapy on mental health and metabolic outcomes in schizophrenia and bipolar disorder: a randomized controlled clinical trial protocol

**DOI:** 10.3389/fnut.2024.1444483

**Published:** 2024-08-21

**Authors:** Calogero Longhitano, Sabine Finlay, Isabella Peachey, Jaymee-Leigh Swift, Flavia Fayet-Moore, Toby Bartle, Gideon Vos, Donna Rudd, Omer Shareef, Shaileigh Gordon, Mostafa Rahimi Azghadi, Iain Campbell, Shebani Sethi, Christopher Palmer, Zoltan Sarnyai

**Affiliations:** ^1^Townsville University Hospital and Health Service, Mental Health Service Group, Queensland Health, Townsville, QLD, Australia; ^2^Laboratory of Psychiatric Neurosciences, Australian Institute of Tropical Health and Medicine, College of Public Health, Medical and Veterinary Science, James Cook University, Townsville, QLD, Australia; ^3^College of Medicine and Dentistry, James Cook University, Townsville, QLD, Australia; ^4^College of Public Health, Medical and Veterinary Sciences, James Cook University, Townsville, QLD, Australia; ^5^Mater Hospital, Aurora Healthcare and James Cook University, Townsville, QLD, Australia; ^6^School of Environmental and Life Sciences, College of Engineering, Science and Environment, University of Newcastle, Callaghan, NSW, Australia; ^7^FoodiQ Global, Sydney, NSW, Australia; ^8^Electrical and Electronics Engineering, College of Science and Engineering, James Cook University, Townsville, QLD, Australia; ^9^Centre for Clinical Brain Sciences, Division of Psychiatry, University of Edinburgh, Edinburgh, United Kingdom; ^10^Metabolic Psychiatry, Department of Psychiatry and Behavioral Sciences, Stanford University School of Medicine, Palo Alto, CA, United States; ^11^McLean Hospital, Harvard Medical School, Belmont, MA, United States

**Keywords:** nutrition, mental health disorders, ketogenic diet, schizophrenia, randomized control trial (RCT), bipolar disorder, dietary intervention, metabolic therapy

## Abstract

**Background:**

Schizophrenia, schizoaffective disorder, and bipolar affective disorder are debilitating psychiatric conditions characterized by a chronic pattern of emotional, behavioral, and cognitive disturbances. Shared psychopathology includes the pre-eminence of altered affective states, disorders of thoughts, and behavioral control. Additionally, those conditions share epidemiological traits, including significant cardiovascular, metabolic, infectious, and respiratory co-morbidities, resulting in reduced life expectancy of up to 25 years. Nutritional ketosis has been successfully used to treat a range of neurological disorders and preclinical data have convincingly shown potential for its use in animal models of psychotic disorders. More recent data from open clinical trials have pointed toward a dramatic reduction in psychotic, affective, and metabolic symptoms in both schizophrenia and bipolar affective disorder.

**Objectives:**

to investigate the effects of nutritional ketosis via a modified ketogenic diet (MKD) over 14 weeks in stable community patients with bipolar disorder, schizoaffective disorder, or schizophrenia.

**Design:**

A randomized placebo-controlled clinical trial of 100 non-hospitalized adult participants with a diagnosis of bipolar disorder, schizoaffective disorder, or schizophrenia who are capable of consenting and willing to change their diets.

**Intervention:**

Dietitian-led and medically supervised ketogenic diet compared to a diet following the Australian Guide to Healthy Eating for 14 weeks.

**Outcomes:**

The primary outcomes include psychiatric and cognitive measures, reported as symptom improvement and functional changes in the Positive and Negative Symptoms Scale (PANSS), Young Mania Rating Scale (YMS), Beck Depression Inventory (BDI), WHO Disability Schedule, Affect Lability Scale and the Cambridge Cognitive Battery. The secondary metabolic outcomes include changes in body weight, blood pressure, liver and kidney function tests, lipid profiles, and markers of insulin resistance. Ketone and glucose levels will be used to study the correlation between primary and secondary outcomes. Optional hair cortisol analysis will assess long-term stress and variations in fecal microbiome composition. Autonomic nervous system activity will be measured via wearable devices (OURA ring and EMBRACE wristband) in the form of skin conductance, oximetry, continuous pulse monitoring, respiratory rate, movement tracking, and sleep quality. Based on the encouraging results from established preclinical research, clinical data from other neurodevelopment disorders, and open trials in bipolar disorder and schizophrenia, we predict that the ketogenic metabolic therapy will be well tolerated and result in improved psychiatric and metabolic outcomes as well as global measures of social and community functioning. We additionally predict that a correlation may exist between the level of ketosis achieved and the metabolic, cognitive, and psychiatric outcomes in the intervention group.

## 1 Introduction

Schizophrenia, schizoaffective disorder, and bipolar disorder are severe and enduring psychiatric conditions characterized by a lifelong pattern of emotional, behavioral, and cognitive symptoms (1–4). These conditions, albeit diagnostically distinct, share several clinical and epidemiological traits, including significant metabolic co-morbidity resulting in a reduction in life expectancy, ranging from 13 to 15 years (5) and up to 25 years in low-income countries (6, 7). Those suffering from chronic psychotic disorders endure the additional burden of poor metabolic health (8). Unfortunately, the currently available antipsychotic medications are only partially effective in assisting with symptom management and come with serious side effects, including diabetes, weight gain, and high blood pressure, leading to a greater prevalence of metabolic syndrome and cardiovascular disorders (9).

**Figure 1:**
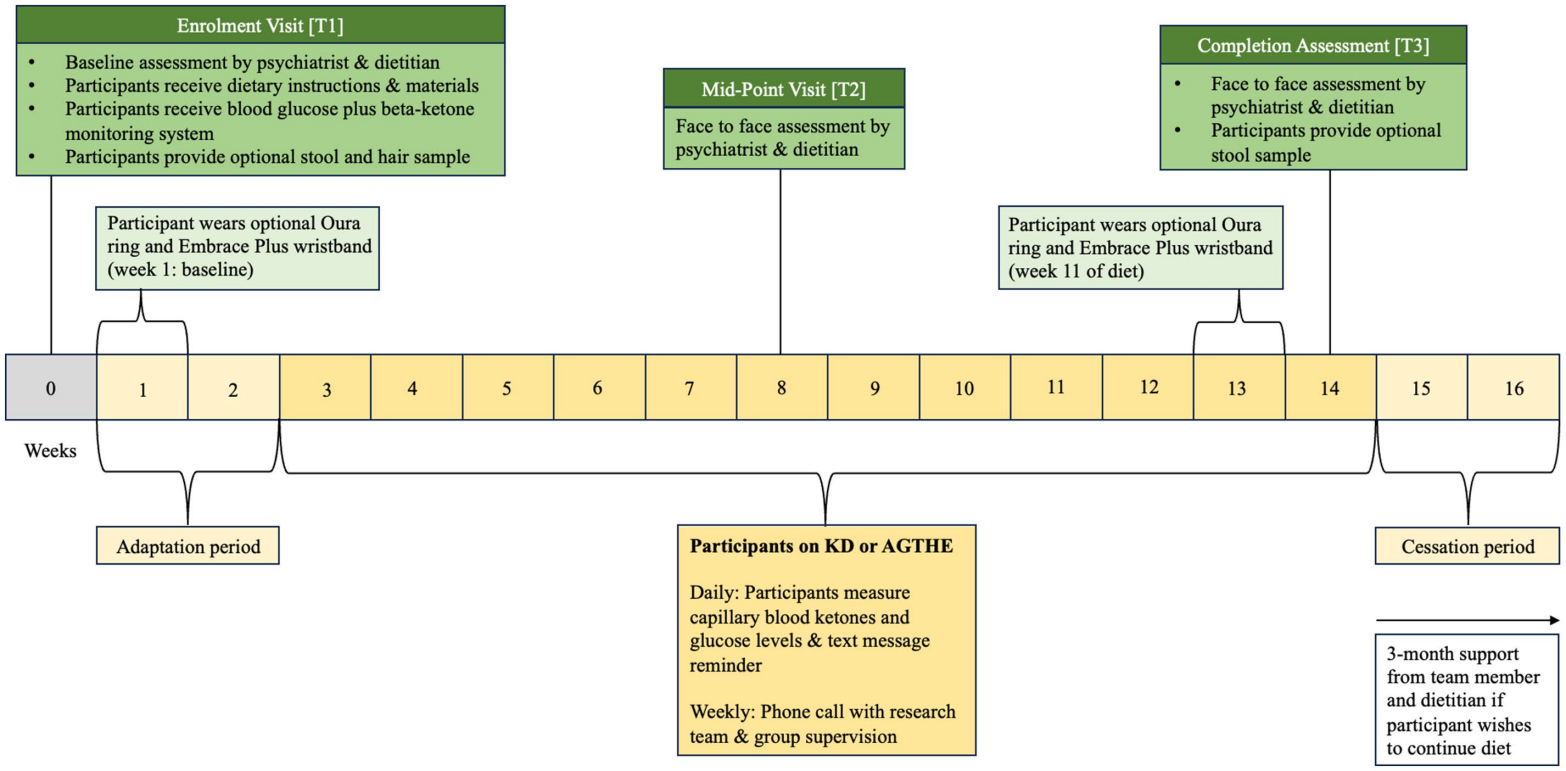
Following Enrolment at T1, participants progress to an “adaptation period” of 2 weeks followed by 6 weeks of allocated dietary intervention until they reach the Mid-Point Visit [T2]. Participants will continue through for further 6 weeks to the Completion Assessment [T3] at week 14.

A major roadblock to developing better therapeutical approaches in serious mental illnesses, such as schizophrenia and bipolar disorder, is the lack of sufficient development in our understanding of disease mechanisms and the identification of potential new therapeutic targets. Since the serendipitous discovery of antipsychotics and lithium in the treatment of schizophrenia and bipolar disorder, respectively, the dominant mechanistic explanations have focussed mainly on major neurotransmitter systems, such as the dopaminergic, serotonergic, and glutamatergic neurotransmission (2, 4, 10). Targeting these systems by pharmaceutical agents has produced major improvements in managing key symptoms and the patient’s quality of life. However, they have also contributed to the development of clinically significant side effects, including motor, metabolic, and endocrine disturbances, which strongly and negatively influence quality of life and life expectancy (11). Those shortcomings of traditional pharmacological approaches have in turn driven recent efforts to identify underlying disease mechanisms as putative targets for symptom improvement and better quality of life for individuals with schizophrenia and bipolar disorder. The development of human genetics and the different-omics technologies, including genomics, transcriptomics, and metabolomics, have revealed the potentially interlinked role of inflammatory/immune mechanisms and altered systemic and brain bioenergetics in the pathophysiology of these disorders (12–16) (Figure 1). Here we briefly review the basics of brain bioenergetics and its alterations in schizophrenia and bipolar disorders to provide a strong mechanistic rationale for our clinical trial.

**Figure 2:**
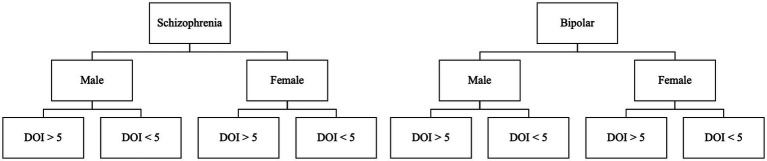
Participants will be stratified to one of 8 groups, depending on their primary diagnosis, biological sex, and duration of illness greater or less than 5 years.

Although constituting just 2–3% of the total body weight, the adult human brain is responsible for the consumption of a disproportionately large fraction, nearly 20%, of the human body’s basal metabolic rate (17). Glucose is the main energy substrate in the brain (18). The high-energy molecule adenosine triphosphate (ATP) is produced from glucose through glycolysis, the non-oxidative breakdown of glucose to pyruvate and lactate in the cytoplasm, through the tricarboxylic acid (TCA) cycle and oxidative phosphorylation (OXPHOS) in the mitochondria (18). Reversing ion movements that generate neuronal post- and pre-synaptic responses consume most of the energy from ATP (19). Glucose is not only the major source of ATP but is also used for the biosynthesis of ribose to form ribonucleic acids, fatty acids, and cholesterol (Figure 2). In addition, glucose through the pentose-phosphate pathway (PPP) is involved in the protection against oxidative stress and through the TCA cycle in the production of amino acids such as glutamate and subsequently GABA (18). Neurons and glia cells cooperate in their energy production. Astrocytes take up glucose from the brain capillaries and metabolize it to lactate through glycolysis, which is transported to neurons through monocarboxylate transporters, a process called the astrocyte-neuron lactate shuttle (19). Neurons then convert lactate to pyruvate and generate ATP thought the TCA cycle and the OXPHOS (18). However, neurons are able to generate ATP directly through glycolysis, even in the presence of sufficient oxygen concentration (aerobic glycolysis), during heightened activity (18). Therefore, deficits in glucose and energy supply in neurons or in glia cells can impair the dynamic regulation of key brain circuits, ultimately resulting in abnormal brain function and behavior (20).

Systemic glucose metabolism abnormalities marked by hyperglycemia and insulin resistance have long been noted in schizophrenia, predating the advent of antipsychotic agents and in treatment-naïve, first-episode psychosis patients (21) In the brain, impairments in glucose metabolism and mitochondrial functions have been detected through diverse methodologies. Essentially, reduced expression of genes encoding various glycolytic enzymes, including the rate-limiting hexokinase, have been observed, alongside diminished levels of enzyme proteins and their activity (22), specifically in glutamatergic neurons in the dorsolateral prefrontal cortex in people with schizophrenia (23, 24). Furthermore, multiple mitochondrial impairments, including altered expression of electron transport chain enzymes, such as Complex-I and Complex-V (ATP-synthase), have been demonstrated in the brains of people with schizophrenia (25–27). Brain imaging studies have identified decreased glucose utilization in the frontal cortex (hypofrontality) and a switch to more glycolytic ATP production associated with elevated brain lactate levels (28, 29). These findings contribute to the conceptualization of schizophrenia as a disease of impaired brain bioenergetics (14).

Similarly, metabolomics and magnetic resonance spectroscopy indicate that energy dysregulation is a central feature of bipolar disorder pathophysiology (30–32). Accordingly, mania represents a condition of heightened cerebral energy metabolism facilitated by hyperglycolysis and glutaminolysis. When oxidative glucose metabolism becomes impaired in the brain, neurons can utilize glutamate as an alternative substrate to generate energy through oxidative phosphorylation. It has been hypothesized that the upregulation of glycolysis and glutaminolysis in this manner causes the brain to enter a state of heightened metabolism and excitatory activity which may underlie the subjective experience of mania (30). Supporting this hypothesis, recent studies have identified mitochondrial abnormalities and resulting elevation of deleterious free oxygen radicals in bipolar disorder (33–35). Taken together, recent progress in the neurobiological understanding of schizophrenia and bipolar disorder points toward the mechanistically important role of abnormal brain bioenergetics in their pathophysiology.

Ketogenic metabolic therapy, achieved via the consumption of a low carbohydrate (CHO)-high fat-containing diet (ketogenic diet; KD), elevates circulating ketone bodies, such as acetoacetate and beta-hydroxybutyrate. Ketone bodies can serve as an alternative energy source for impaired glucose metabolism and support mitochondrial function (36), thus potentially counteracting the underlying bioenergetic abnormalities in schizophrenia and bipolar disorder (37, 38). Three weeks of KD or beta-hydroxybutyrate administration effectively normalized behavioral impairments in a hypo-glutamatergic animal model of schizophrenia (39–41). After highly encouraging results from case studies showing dramatic improvements in a wide range of symptoms and quality of life in patients with schizophrenia on KD (42–45), a recent single-arm clinical trial in which patients with schizophrenia and bipolar disorder consumed a medically supervised KD, showed improvements in several psychiatric symptoms and metabolic functions (46). KD was also successfully used in a recent single-arm trial in patients with bipolar disorder (47). To indicate an increasing interest in ketogenic therapeutic interventions in psychiatry a recent pilot trial protocol aims to investigate the effect of a ‘ketogenic-mimicking diet’ (combining supplementation of ketone esters with a low glycemic index dietary intervention) on neural network stability, mood, and biomarker outcomes in the setting of bipolar disorder (48).

Taken together, positive results from extant preclinical studies, case studies, and uncontrolled clinical trials point toward a clinically significant role for ketogenic metabolic therapy in the treatment of serious mental illness and offer hope to millions of individuals worldwide, who suffer from those conditions. However, no data from randomized controlled clinical trials exist to date. Such clinical trial ought to compare the effects of KD with another dietary intervention under circumstances that are identical for all randomly assigned trial participants in every possible aspect, such as trial management, clinician’s attention, and outcome measures, to establish the specific contribution of the ketotic metabolic state on the outcomes of interest. Here we report the detailed protocol of a randomized controlled clinical trial to investigate the efficacy of ketogenic metabolic therapy on psychiatric, cognitive, and functional symptom improvement and metabolic changes in schizophrenia and bipolar disorder.

## 2 Methods

### 2.1 Study design

This randomized placebo-controlled parallel-designed clinical trial is registered with the Australian New Zealand Clinical Trials Registry (ANZCTR), the mandated online register of clinical trials being undertaken in Australia, New Zealand, and other Oceanic Nations (Reg. No: ACTRN12623000854639).

#### 2.1.1 Recruitment and screening

Advertisements will be disseminated to local community mental health teams, general practitioners, general hospitals, and other health facilities. Advertisement is also broadcast via social media networks and press launches from Townville University Hospital and James Cook University. Prospective participants register via the trial website^1^ and will then receive an email with information material relating to the structure and requirements of the trial. Prospective participants will then be screened for eligibility via telephone by the principal investigator, who is a senior psychiatrist. During the screening call or Timepoint 0 [T0], the principal investigator (CL) will check the prospective participant’s eligibility to enroll in the trial against the inclusion and exclusion criteria (Table 1).

**Table 1:** Participant eligibility.

Inclusion criteria	Exclusion criteria
To be eligible for this study, an individual must meet the following criteria: Have been diagnosed by a psychiatrist with either schizophrenia, schizoaffective disorder, or Bipolar Affective Disorder and have had the condition for at least 6 months. Being clinically stable (no episodes of hospitalization or significant treatment changes for at least 3 months) Possess the mental capacity to provide informed consent and willingness to sign a written informed consent document. Aged ≥18 years old. Able to understand the basic principles of the specific diet and follow dietary instructions as provided by the study dietitian. Willingness to adhere to all study procedures including the completion of an accurate diet and symptoms diary, daily blood glucose and ketone monitoring, and to attend the visits as scheduled.	Individuals who meet any of the following criteria will be excluded from participation in this study: Pregnant, breastfeeding, or planning to become pregnant within 3 months. Active substance misuse with alcohol or illicit drugs. Use of the ketogenic diet in the previous 2 months. Currently following a vegan diet. Admission to a mental health hospital within the past 3 months. Inability to complete visits and assessments. Uncompensated cardiovascular disease. Severe hyperlipidemia. Type 1 diabetes. History of eating disorder. BMI < 18.5 kg. On medication that can cause ketosis. Not willing to change diet or unable to change diet due to medical reasons. Active liver or kidney disease. No access to cooking facilities and ingredients to prepare recipes following the specific diet. Current involvement in another research study.

#### 2.1.2 Assessments

Eligible participants progress to the first physical visit (Enrolment Visit at Timepoint 1 [T1]) and are asked to complete a three-day food diary before attending the appointment. During the enrolment visit [T1], formal written consent is obtained by a trial psychiatrist (CL, SG, or OS) before baseline assessments of psychiatric, cognitive, and metabolic outcome measures are taken. Participants are then randomized into either the Ketogenic Diet (KD) or the Australian Guide to Healthy Eating (AGHE) group by stratified, computer-generated blinded allocation. Following randomization, participants progress to the dietitian assessment and group-specific dietary education. Dietary education and counseling for each group are provided by a senior dietitian, including the provision of the existing evidence base for nutrition in mental health to assist in personalizing the KD or AGHE diet. Consideration will be given to their metabolic health, dietary preferences, age, sex, and physical activity level that may impact their estimated energy requirements (EER). Participants will then receive detailed dietary instructions and specific educational material to foster independent meal preparation. All participants, irrespective of their group allocation, will be instructed to complete a 2-week diet “adaptation period” where both groups progressively adjust their diet to minimize any side effects due to metabolic adaptation to the new diet and to allow participants to adjust their daily routine to the new food preparation requirements. Participants will be asked to complete daily food diaries and are provided with weekly dietetic support in the form of face-to-face consultation, and phone-, and email follow-up to increase compliance, alter individual diet prescriptions where required, and for safe cessation of the diet. In the second visit (Mid-Point Visit or Timepoint 2 [T2]), psychiatric and metabolic assessments are repeated. In the third visit (Completion Assessment or Timepoint 3 [T3]), all psychiatric, metabolic, and cognitive assessments are repeated. T1 is at baseline (week 0), T2 occurs on week 8, and T3 on week 14. The intervention is 12 weeks from week 3 to week 14, plus a 2-week washout period, for a total of 14 weeks duration (Figure 1). During the washout period, participants are instructed to progressively revert to a diet of their preference. Participants may also continue with their allocated diet, if they choose to do so.

Pre-existing psychiatric and metabolic treatment will continue as per the treating team’s medical advice. Metabolic medication might be adjusted for those in the KD group in collaboration with their physician to minimize the risk of hypoglycemia. Participants in both groups will be given a blood ketone and glucose monitoring device at the end of the enrolment visit (T1) and instructed to take a daily capillary blood sample to measure glucose and ketone levels. The measurement will occur consistently every day, 2 h before their routine dinner time. Daily glucose and ketone measurements are uploaded to a team’s database daily and strictly monitored to detect any emergent metabolic abnormality.

#### 2.1.3 Allocation

Participants will be blinded to the study hypothesis and interact with clinicians who are kept blind to their group allocation as far as possible. Allocation will occur only after the initial psychiatrist assessment. If a psychiatrist becomes accidentally unblinded to a participant’s allocation during the running of the trial, a different team psychiatrist will be asked to continue the remaining time points assessment(s) for that participant. Participants in both groups will be treated identically in all respects except for the intervention being tested.

The study is being conducted at a single site at the Translational Research Facility, Australian Institute of Tropical Health and Medicine, James Cook University at Townsville Campus, Queensland, Australia. Human Research Ethics Approval (HREC/2022/QTHS/85408) for this study was received by the Townsville University Hospital HREC board, responsible for the study population’s catchment area.

### 2.2 Experimental intervention

The intervention involves a 12-week dietitian-led, medically supervised KD, designed to induce a state of nutritional ketosis. Several dietary regimes for achieving ketosis in adults exist including the Modified Atkins Diet (MAD)/ Modified Ketogenic Diet (MKD), Low Glycaemic Index Treatment (LGIT), and the Classical Ketogenic Diet (Table 2). These diets share a common principle of reducing carbohydrate (CHO) intake while increasing fat consumption. The variation in the degree of CHO reduction and fat increase distinguishes these regimes. In this trial, a MKD (2:1 ratio) will be implemented to induce ketosis in participants. The MKD was selected for its higher compliance rates (49, 50) and its allowance for greater flexibility and food variety compared to the classical ketogenic diet (4:1 ratio). The prescribed amount of macronutrient distribution will aim for approximately 75% of EER from fat, 5% from CHO, and the remaining from protein. Additionally, participants following the MKD will be recommended to supplement with a CHO-free multivitamin in accordance with best practice guidelines (49).

**Table 2:** Most commonly used therapeutic Ketogenic diets.

	Modified Atkins diet	Modified Ketogenic diet	Low glycaemic index treatment	Classic Ketogenic diet
Fat (%)	65	75	60	90
Carbohydrates (%)	10	5	10	4
Protein (%)	25	20	30	6

### 2.3 Control diet

The study’s control group adheres to a 12-week regimen of dietitian-led and medically supervised nutrition, aligning with the principles outlined in the Australian Guide to Healthy Eating (AGHE). Macronutrient intake, including carbohydrates, fat, and protein, is calculated to match individual factors such as age, weight, and level of physical activity as set out in the guidelines. Within this framework, the recommended optimal macronutrient ranges from the AGHE are encouraged, with protein comprising 11%, fats ranging from 20 to 35%, and the remainder from carbohydrates (see Table 3) (51).

**Table 3:** Ketogenic diet vs. the Australian guide to healthy eating.

Ketogenic diet	Australian guide to healthy eating
< 50 g of carbohydrates per day Total daily calories: 70–80% fat 5–10% carbohydrates 10–20% protein	Plenty of vegetables of different types and colors, and legumes/beans Fruit Grain (cereal) foods, mostly wholegrain and/or high cereal fiber varieties, such as breads, cereals, rice, pasta, noodles, polenta, couscous, oats, quinoa, and barley Lean meats and poultry, fish, eggs, tofu, nuts and seeds, and legumes/beans Milk, yogurt, cheese, and/or their alternatives, mostly reduced fat

## 3 Psychiatric outcome measures

### 3.1 Positive and negative syndrome scale

Positive and Negative Syndrome Scale (PANSS) is among the most-validated instruments for assessing positive, negative, and general psychopathology associated with schizophrenia. The PANSS is a standardized clinical interview that rates the presence and severity of positive and negative symptoms, as well as general psychopathology for people with schizophrenia within the past week. Of the 30 items, seven are positive symptoms, seven are negative symptoms, and 16 are general psychopathology symptoms. Symptom severity for each item is rated according to which anchoring points in the 7-point scale (1 = absent; 7 = extreme) best describe the presentation of the symptom (52). Positive and negative scales showed good inter-rater reliability and interclass correlation coefficients (ICC) of 0.72 and 0.80, respectively. Inter-rater reliability was moderate for the general psychopathology scale; ICC = 0.56 (53).

### 3.2 The young mania rating scale

The Young Mania Rating Scale (YMRS) is a clinical interview scale and is one of the most frequently utilized rating scales to assess the severity of manic states in bipolar affective disorder. The scale has 11 items and is based on the patient’s subjective report of his or her clinical condition over the previous 48 h. Additional information is based on clinical observations made during the clinical interview. The items are selected based on published descriptions of the core symptoms of mania. The YMRS follows the style with each item given a severity rating. Four items are graded on a 0 to 8 scale (irritability, speech, thought content, and disruptive/aggressive behavior), while the remaining seven items are graded on a 0 to 4 scale. These four items are given twice the weight of the others to compensate for poor cooperation from severely ill patients (54).

### 3.3 World health organization disability assessment schedule, version 2

The World Health Organization Disability Assessment Schedule, version 2 (WHODAS 2.0) is the standard measure of disability promoted by the World Health Organization. WHODAS 2.0 is a patient-reported outcome instrument that uses 36 questions to assess the global health status of patients across 6 health domains, i.e., Cognition, Mobility, self-care, social interaction, life activities, and social participation. The WHODAS 2.0 is a validated and established questionnaire that can be used to assess the health status of patients irrespective of disease (55).

### 3.4 The Beck depression inventory

The Beck Depression Inventory (BDI) is one of the most widely used measures in both research and clinical practice for assessing depression. BDI is a 21-item, self-report rating inventory that measures characteristic attitudes and symptoms according to diagnostic criteria listed in the Diagnostic and Statistical Manual for Mental Disorders. BDI demonstrates high internal consistency, with alpha coefficients of 0.86 and 0.81 for psychiatric and non-psychiatric populations, respectively (56).

### 3.5 The affective lability scale 18

The Affective Lability Scale 18 (ALS-18) is a valid and reliable instrument for measuring affect lability. ALS measures individual proneness to rapid shifts from the different emotional states of anxiety, depression, anger, and hypomania (57). The ALS-18 item version is based on a three-factor model of affective lability (anxiety/depression, depression/elation, and anger), with each factor retaining at least two items from each of the original six subscale versions. ALS-18 is highly correlated with the original 54-item version (*r* = 0.94).

## 4 Assessment of cognitive function

Cognitive functioning at baseline (T0) and post-intervention (week 14, [T3]) will be assessed using the Cambridge Neuropsychological Test Automated Battery (CANTAB). CANTAB measures are a reliable, valid, sensitive way of collecting comprehensive and accurate information on cognitive functioning in a clinical sample (58). Test batteries are delivered digitally using an iPad, allowing for standardized data collection at multiple time points and automated creation of comprehensive data sets. The level of information provided by each CANTAB measure allows for in-depth analysis of highly specific components of cognitive functioning providing specific insight into intervention efficacy within the context of a much wider dataset (59, 60). A customized test battery, consisting of 7 separate neuropsychological assessments (Table 4) was designed for data collection during this clinical trial.

**Table 4:** CANTAB neuropsychological test aspects.

Neuropsychological test (CANTAB)	Test variation	Administration time (Min)	Outcome measures
Motor Screening Task (MOT)	Voice	2	Accuracy Difficult/speed adjustment
Spatial Working Memory (SWM)	Recommend Standard Tone 2.0	4–6	Errors Incorrect selection, reselection
Emotional Recognition Task (ERT)	Short (Caucasian)	6–10	Correct recognition Response latency
Rapid Visual Processing (RVP)	1 Target Tone	7	Response latency Probability of false alarms sensitivity
Paired Associated Learning (PAL)	Recommended Standard Tone	8	number of errors number of trails required memory scores stages completed
One Touch Stocking of Cambridge (OTS)	Short	10	number solved on first choice Mean number of choices to solve Mean latencies
Cambridge Gambling Task (OTS)	Ascending First Shortened Tone	12–18	Risk-taking Decision-making quality Decision time Risk adjustment Delay aversion Impulsivity
Total administration time		49–61	

## 5 Clinical laboratory measures

*Anthropometric biomarkers*: Bodyweight will be measured using an electric scale (two decimal accuracies), while the standing height will be measured by a stadiometer at baseline, week 8 and week 14. The body mass index (BMI) will be calculated using these measures.

*Cardiovascular biomarkers*: At each visit, blood pressure will be measured using an Omron HEM7 120 Blood Pressure Monitor. Blood pressure will be measured from the left arm while the participant is seated and at rest. Two readings will be collected for systolic and diastolic blood pressure. Heart rate will likewise be assessed at a resting position using the Omron HEM7 monitor.

*Metabolic biomarkers*: Fasting peripheral blood will be collected in participants at baseline, week 8, and week 14. 0.5 mL of whole blood will be transferred from the EDTA tube to a new tube and frozen until analysis. The remaining samples will be spun at 3000 rpm for 10 min. Serum and Plasma will be transferred to new tubes and frozen at −80 degrees Celsius until analysis. From the samples, the metabolic biomarkers: triglycerides, high-density lipoprotein (HDL-c), low-density lipoprotein (LDL-c), total cholesterol (TC), insulin, glucose, and HbA1c will be measured.

*Immune biomarkers*: Interleukin-6 (IL-6), IL-12, tumor-necrosis-factor (TNF) alpha, albumin, fibrinogen, and c-reactive protein (CRP) will be assessed using the collected blood.

*Neuroendocrine biomarkers*: Dehydroepiandrosterone sulfate (DHEAS) will be assessed from the collected blood samples, while cortisol concentrations will be assessed from hair samples collected at baseline and week 14 using the Salimetrics Saliva Cortisol ELISA kit.

## 6 Randomization

For this study, stratified randomization will be used. We will stratify by assuming eight groups based on the following criteria: diagnosis (schizophrenia or bipolar disorder), gender (male or female), and duration of illness (DOI; more or less than 5 years). Groups will be divided as demonstrated in Figure 2.

Each of the stratified groups will be randomly allocated into either KD or AGHE. The allocation will be concealed from the investigators and will only be known by the Clinical Research Coordinator. Each subsequent entry to each stratum will be sequentially followed, thereby creating a stratified, random allocation. Recruitment for this trial will be continuous for 18 months or until the required participation number has been reached.

## 7 Measurement tools used

### 7.1 Wearable devices

An Oura ring (Ōura, Oulu, Finland) and an Embrace wristband (Empatica, Cambridge, United States) will be offered to all participants to wear for a week during the introductory period before starting the diet (week 1 [T1]) and during week 14, just before finishing the diet. This measurement is optional and can be selectively opted out by the participant. Sensor biomarker data including heart rate (HR), heart rate variability (HRV), and electrodermal activity (EDA) from the Oura and Embrace devices will be utilized to provide an overall autonomic nervous system (ANS) stress score, using a pre-trained machine learning model previously shown to be a reliable predictor of acute stress (61).

### 7.2 Stool collection

It will be optional for all participants to supply a stool sample at the start of the trial (prior to T1) and again at the end of the trial period (week 14) for the future analysis of changes in the fecal microbiome composition and its metabolites. The participant will be supplied with a self-collection kit during the Week 0 assessment, and they can collect the specimen at home at their earliest convenience. The kit can be posted to the research team.

### 7.3 Hair collection

It will be optional for all participants to supply a hair sample at the start of the trial (prior to T1) and again at the end of the trial period (week 14) for future analysis of cortisol to assess the cumulative impact of the biological stress response over time. The small sample (5 mg) will be taken by a researcher during the physical assessment.

### 7.4 Glucose and ketone measurement

Ketone and glucose levels will be monitored by the participants from home. A “blood glucose plus beta-ketone monitoring system” and test strips will be supplied to all participants. The participant will be required to take daily samples by finger prick (1.0 uL blood) 2 h before eating dinner. The monitor system will inform the investigators and the participants about the time spent in ketosis. During the week 0 assessment, participants will be asked a preference to inform us about their daily ketone and glucose results. This may also include a daily reminder text to the participant if they prefer. The participant can choose to either upload a photo of the result to a phone used during the trial, text-message the result to the phone or they can choose to receive a daily phone call and talk to a team member. The trial phone will be monitored by a team member 24/7.

## 8 Data analysis

### 8.1 Sample size and statistical power

The optimal sample size is calculated based on a statistical power of 90% and a significance level of 0.05 (two-tail). To calculate the sample size, we have estimated the incidence of significant improvement in primary or secondary outcome measures to be 75% in group 1 (individuals on the KD) and 40% in group 2 (individuals on the AGHE diet). We will aim for a 1:1 enrolment ratio.

Using these values, our estimated sample size is 80 individuals (40:40), however, considering a dietary intervention attrition rate of at least 20%, we will aim to recruit 100 individuals (50:50).

### 8.2 Data analysis plan

The analysis of the biological samples will be conducted by biomedical staff at James Cook University overseen by the James Cook University Specialist in Clinical Biochemistry and Clinical Research Coordinator of the Australian Institute of Tropical Health and Medicine. This will follow standard analytic pipelines established in these units.

We predict that analyses of observed data will be largely descriptions of response, adherence, and distributions of values. Simple correlations between study parameters will follow standard statistical methods and the assistance of a statistician has been sought to define the statistical plan before the research plan is finalized. Software packages will include SPSS (v28) and *R* (v4.2.1).

In line with Burgess et al. (62) and many other modern statisticians who have described the procedure of hypothesis testing on baseline characteristics as unnecessary and potentially harmful (63), we will present the distribution of baseline information of intervention groups in a simple table, allowing readers to compare the extent of similarities, each with a mean and percentage value for both treatment groups separately. Additional co-variables may likely be at play, especially socioeconomic status, BMI, and severity of illness. Therefore, a degree of covariate adjustment is expected at the statistical calculation stage. Logistic regression and Analysis of Covariance are likely going to be incorporated into the data analysis plan.

For our quantitative endpoints (e.g., on psychiatric scales and metabolic parameters), linear mixed effects (LME) models will be used to compare the intervention and control groups. If normality cannot be assumed, a logarithmic transformation of the response variable will be performed to meet the assumption of normality. Mixed-effect logistic regression will be used for binary endpoints and longitudinal data. If data normality cannot be achieved, a comparison of our variables of interest between groups will take place using non-parametric tests, e.g., the Mann–Whitney U test. All analyses will be conducted by an intention-to-treat (ITT) approach using all available data, including those who were not to complete the study. Patient data will be analyzed per their original treatment allocation. All data analyses will be performed using 5% significance levels and audited by several members of the research team to ensure accuracy before submission.

## 9 Discussion

The rationale for this randomized controlled clinical trial is based on a converging line of evidence coming from recent studies showing a variety of brain bioenergetic abnormalities in schizophrenia and bipolar disorders, prior preclinical research demonstrating the efficacy of the ketogenic diet in animal models of schizophrenia, as well as recent encouraging results from case studies and pilot, single-arm clinical trials that indicate clear symptom improvement and better metabolic control in patients with serious mental illness on a ketogenic diet. We expect that the dietary metabolic interventions in our trial will result in beneficial changes in symptoms, everyday functioning, and overall metabolic health for our participants. The control diet, based on the AGHE guidelines, is likely superior in terms of its health effects compared to the participants’ usual dietary patterns because of its focus on incorporating whole grains, dietary fiber, plant-based proteins, and unprocessed food. We expect that the ketogenic metabolic therapy, with its targeted effect on brain and systemic energy metabolism, will result in further improvements in metabolic health, controlling psychiatric symptoms and improving overall functioning, above and beyond the control diet. In addition to establishing efficacy, our design will allow the assessment of the participant’s compliance with and adherence to a relatively restrictive dietary schedule that requires major deviations from their usual diet. This will be important for future dietary metabolic interventions in serious mental illness, where patients are generally perceived to be struggling with such challenges.

In case of demonstrated efficacy, it is crucial to understand the mechanism of action of this novel metabolic intervention to give informed advice to the psychiatric community on any potential change in clinical practice. Our trial will collect data from different domains, including systemic metabolism and inflammatory processes, autonomic nervous system activity, sleep patterns, and changes in the gut microbiome. Outcome measures from these systems, when correlated with improved psychiatric symptoms, cognitive functions, and overall daily functioning will inform us about the potential involvement of underlying mechanisms. With regards to mechanisms of action of dietary intervention, the possible mediating role of the gut microbiome is of primary importance as the ecosystem of the bacteria in the gastrointestinal system interacts with the consumed diet even before the different nutrients and other food-derived molecules get into the bloodstream (64–67). It is conceivable that some of the effects of the ketogenic metabolic therapy may be mediated by the gut microbiome, either directly through microbiome-derived metabolites that reach the brain or indirectly through changes in the enteric nervous system activity. We aim to establish such a role for the gut microbiome by demonstrating ketogenic diet-related changes associated with symptom improvement. Ketone bodies, beta-hydroxybutyrate, and acetoacetate are the metabolic products of the liver while on the ketogenic diet (36). Ketones have been shown to directly benefit brain bioenergetics (68, 69). The daily ketone monitoring will make it possible not only to confirm adherence to the ketogenic diet but also to correlate ketone levels with daily changes in the self-reported energy levels of the participants. Alterations in autonomic nervous system activity and sleep have been widely reported in schizophrenia (70) and bipolar disorders (71, 72). The ketogenic diet seems to be effective in influencing these functions. Therefore, the potential mechanistically important role of autonomic nervous system activity and sleep health can be uncovered by monitoring these functions using wearable devices before the introduction and at the end of the dietary intervention. Although human studies are rarely designed to include an experimental intervention that directly informs about exact mechanisms of action, the complex analysis of multiple, interacting systems will show biomarkers that are specifically modified by the treatment and associated with improvements in different symptom domains.

Our trial design has some limitations. Firstly, despite our best efforts to keep the overseeing psychiatrists blind to the dietary arm allocation of individual participants, we cannot rule out the possibility that some participants may disclose their diets during the psychiatric assessments in the middle or at the end of the trial. Also, an argument can be made that some of our motivated participants may have information about the expected benefits of the ketogenic metabolic therapy in their condition, which could conceivably introduce an expectation bias if assigned to the ketogenic arm of the trial. We will, however, obtain their daily blood ketone levels which can be correlated with the results of the psychiatric and cognitive assessment, and give an objective control measure to address the above limitations. Another possible limitation is that due to the nature of this clinical trial conducted in an outpatient setting, relying on the ability of the participant to adhere to a relatively restrictive dietary regime independently, the recruited participants will be better functioning than some other patients with serious mental illness, resulting in a self-selected study population. We acknowledge this limitation, but we argue that although we might be able to demonstrate better adherence in this self-selected group, it may be a strength of the trial to demonstrate symptom improvement in participants who may be somewhat better functioning already. Additionally, demonstrating adherence to a restrictive dietary regimen by a population with severe mental disorders in the community may in itself be regarded as a satisfactory outcome measure.

In conclusion, we expect this randomized controlled clinical trial to assess the feasibility, efficacy, and safety of ketogenic metabolic therapy in schizophrenia and bipolar disorder. To our knowledge, our proposed trial is the first of its kind worldwide. We hypothesize that this study will reveal improvements in symptoms, overall quality of life, and better metabolic functioning for our participants. Results from this trial may inform future studies on more specific mechanisms of action, as well as introduce a novel treatment modality to manage psychiatric disorders that would otherwise have been considered as long-term, debilitating conditions.

## Data availability statement

The original contributions presented in the study are included in the article/supplementary material, further inquiries can be directed to the corresponding author.

## Ethics statement

The studies involving humans were approved by the Human Research Ethics Committee of Townsville University Hospital and James Cook University [HREC/2022/QTHS/85408 and JCU (James Cook University) C45]. The studies were conducted in accordance with the local legislation and institutional requirements. The participants provided their written informed consent to participate in this study.

## Author contributions

CL: Conceptualization, Data curation, Formal analysis, Funding acquisition, Investigation, Methodology, Project administration, Resources, Software, Supervision, Validation, Visualization, Writing – original draft, Writing – review & editing. SF: Data curation, Methodology, Project administration, Software, Writing – original draft, Writing – review & editing. IP: Data curation, Project administration, Visualization, Writing – review & editing. J-LS: Conceptualization, Data curation, Methodology, Project administration, Supervision, Writing – review & editing. FF-M: Conceptualization, Methodology, Validation, Writing – review & editing. TB: Conceptualization, Funding acquisition, Methodology, Resources, Writing – review & editing. GV: Data curation, Formal analysis, Methodology, Visualization, Writing – review & editing. DR: Conceptualization, Funding acquisition, Project administration, Supervision, Writing – review & editing. OS: Project administration, Writing – original draft, Writing – review & editing. SG: Project administration, Writing – review & editing. MA: Formal analysis, Methodology, Software, Supervision, Writing – review & editing, Conceptualization, Data curation. IC: Conceptualization, Methodology, Resources, Validation, Writing – original draft. SS: Conceptualization, Methodology, Supervision, Validation, Writing – review & editing. CP: Conceptualization, Funding acquisition, Methodology, Supervision, Validation, Writing – original draft. ZS: Conceptualization, Data curation, Formal analysis, Funding acquisition, Investigation, Methodology, Project administration, Resources, Software, Supervision, Validation, Visualization, Writing – original draft, Writing – review & editing.

## Footnotes

^1^ https://nqdit.com.au/
